# Timeliness of Nongovernmental versus Governmental Global Outbreak Communications

**DOI:** 10.3201/eid1807.120249

**Published:** 2012-07

**Authors:** Luke Mondor, John S. Brownstein, Emily Chan, Lawrence C. Madoff, Marjorie P. Pollack, David L. Buckeridge, Timothy F. Brewer

**Affiliations:** McGill University, Montreal, Quebec, Canada (L. Mondor, D.L. Buckeridge, T.F. Brewer);; Harvard–Massachusetts Institute of Technology, Boston, Massachusetts, USA (J.S. Brownstein, E. Chan);; Children’s Hospital Boston, Boston (J.S. Brownstein, E. Chan);; International Society for Infectious Diseases, Brookline, Massachusetts, USA (L.C. Madoff, M.P. Pollack, T.F. Brewer);; University of Massachusetts, Worcester, Massachusetts, USA (L. Madoff);; and Harvard Medical School, Boston (J.S. Brownstein)

**Keywords:** outbreak reporting, pandemic preparedness, informal sources, emerging infectious diseases, ProMED-mail, nongovernmental, governmental, global, outbreaks, communications

## Abstract

To compare the timeliness of nongovernmental and governmental communications of infectious disease outbreaks and evaluate trends for each over time, we investigated the time elapsed from the beginning of an outbreak to public reporting of the event. We found that governmental sources improved the timeliness of public reporting of infectious disease outbreaks during the study period.

Rapid public communication of incipient disease threats, even with incomplete information, might enable quicker response measures, including enhanced disease surveillance and initiation of protective measures, for those at risk (*1*). Traditionally, public notifications are communicated through a hierarchical infrastructure from which local, provincial or state, and national health authorities obtain information by interacting with health care providers and diagnostic laboratories (*2*). However, many health authorities now rely on informal outbreak-reporting systems, such as ProMED-mail, for timely signals of infectious threats (*3**,**4*), as encouraged by the revised WHO International Health Regulations (*5*). Research has suggested that globally, informal sources provide outbreak warnings faster than traditional governmental reporting mechanisms (*6*). However, existing research of this assertion has been limited to disease-specific evaluations (*7*) or descriptive summaries (*8*). We compared the timeliness of initial outbreak communications cited by nongovernmental sources to those of governmental sources and examined temporal trends in the time from outbreak onset to public communication for outbreaks communicated by each source, independently.

## The Study

The study database consisted of 398 unique human infectious disease outbreak events collected from Disease Outbreak News, published online by the World Health Organization during 1996–2009 (*9*). For each outbreak, we defined the initial source or sources of the first public communication as the individual, organization, or website that first publicly communicated information regarding the disease threat (locally or internationally, orally or in writing). The corresponding date of communication was identified by using outbreak reports disseminated by ProMED-mail (*10*). All outbreaks were categorized as having been first communicated by >1 nongovernmental or governmental source, or simultaneously by both types of sources. When an outbreak was simultaneously first communicated by nongovernmental and governmental sources (n = 5), the outbreak was repeated in the dataset and each source was given credit. This adjustment increased the number of outbreak events to 403.

To characterize the timeliness of outbreak communications, for each reporting source of an event, we calculated the median time in days, and bootstrapped 95% CI, from outbreak start to public communication (Table 1). Median reporting times were calculated for the entire study period (1996–2009), before and after public recognition of severe acute respiratory syndrome (SARS) (March 12, 2003), and for each WHO-defined geographic region. The effect of the initial reporting source on the timeliness of outbreak communication was quantified by using negative binomial regression after adjusting for geographic region and whether the outbreak occurred before or after SARS. These variables were included in the model on the basis of a priori assumptions that public health infrastructure can vary by geographic and political region and that new pandemic preparedness strategies, including use of informal information to initiate public health responses, were developed in response to the SARS epidemic (*11*). Interaction terms between each variable were examined but were not included in the final model because none reached statistical significance (p>0.05). Temporal trends were assessed by using univariate negative binomial regression models, stratified by source category. These models included 1 covariate for the year of outbreak start.

**Table 1:** Time from the estimated start of an outbreak to its earliest communication by source*

Variable	Governmental sources			Nongovernmental sources		p value
	No. outbreaks	Median no. days (95% CI)		No. outbreaks	Median no. days (95% CI)	
Period						
1996–2009	163	33.0 (30–44)		103	23.0 (20–32)	0.200
Pre-SARS	90	39.5 (31–51)		61	29.0 (20–50)	0.161
Post-SARS	73	29.0 (25–37)		42	21.5 (17–32)	0.613
Geographic location						
Africa	85	37.0 (29–51)		41	31.0 (23–57)	0.733
Americas	13	30.0 (21–63)		12	25.0 (20–34)	0.568
Eastern Mediterranean	24	41.0 (23–51)		9	31.0 (16–82)	0.903
Europe	11	31.0 (23–79)		9	20.0 (13–184)	0.909
Southeast Asia	8	28.0 (10–62)		11	14.0 (11–51)	0.431
Western Pacific	22	26.0 (12–52)		21	18.0 (13–33)	0.789

*Bootstrapping with 10,000 replicates was used to calculate 95% CIs for median values. SARS, severe acute respiratory syndrome.

**Figure 1:**
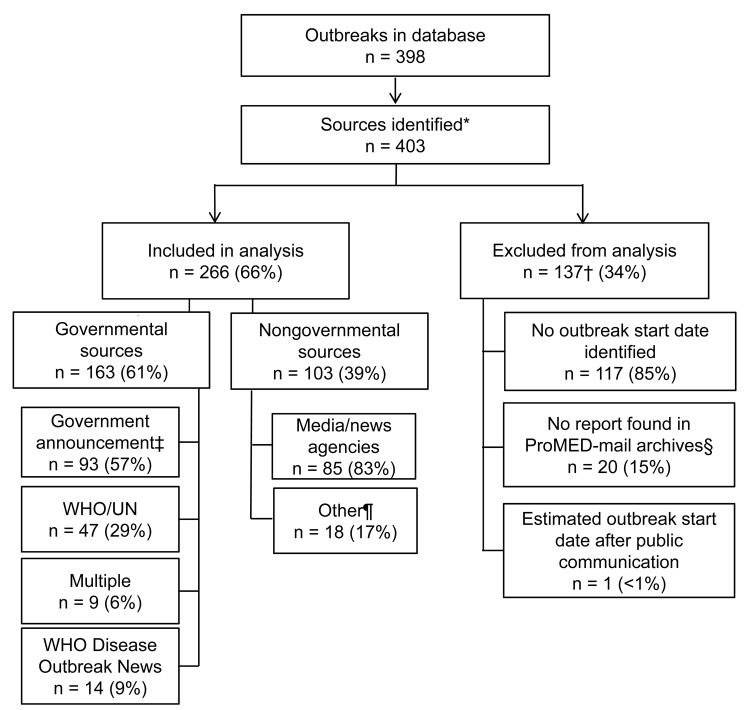
Exclusion criteria applied to database of 398 outbreak events publicly reported through the World Health Organization (WHO) Disease Outbreak News during 1996–2009 and breakdown of nongovernmental and governmental sources used to compare the timeliness of outbreak communications. UN, United Nations. *More than one source may be identified for a given outbreak; †categories for exclusion are not mutually exclusive; ‡health officials, ministries of health, laboratories, hospitals, etc.; §included in sensitivity analysis; ¶includes nongovernmental organizations, individual accounts, ProMED requests for information, and multiple sources.

Of all initial outbreak reports identified, 137 were excluded from analysis for ≥1 of the following reasons (Figure 1): 117 (85%) of the excluded reports were missing information on the estimated outbreak start date; 20 (15%) were not found in the ProMED-mail archives; and 1 (1%) outbreak estimated start date occurred after the date of public communication of the outbreak. Of the 266 (66%) outbreaks included in analysis, 163 (61%) were first publicly communicated by governmental sources, and 103 (39%) were first communicated by nongovernmental sources. Chi-square tests showed no significant differences in the proportions of governmental and nongovernmental sources included in the analysis versus those excluded (p = 0.315).

The median time from estimated outbreak start to initial public communication was 10 days shorter for nongovernmental sources (23 days, 95% CI 20–32) than for governmental sources (33 days, 95% CI 30–45), although this difference was not significant according to the Wilcoxon rank-sum test (p = 0.200) (Table 1). Additionally, multivariate modeling showed no significant difference after covariates were adjusted for (incidence rate ratio [IRR] 0.95, 95% CI 0.77–1.18) (Table 2). The effect of missing data was assessed in sensitivity analyses for all outbreaks for which we had an estimated outbreak start date (17 of 20). When we used the WHO Disease Outbreak News communication date, our results did not change when crediting either governmental sources (IRR = 0.88, 95% CI 0.71–1.09) or nongovernmental sources (IRR = 1.086, 95% CI 0.882–1.336).

**Table 2:** Comparison of the timeliness of outbreak communications by nongovernmental and governmental sources*

Variable	IRR (95% CI)	p value
Source		
Governmental	Ref	
Nongovernmental	0.950 (0.765–1.180)	0.645
Chronological order		
Pre-SARS	Ref	
Post-SARS	0.713 (0.576–0.884)	0.002
Geographic location		
Africa	Ref	
Americas	0.773 (0.531–1.126)	0.180
Eastern Mediterranean	0.912 (0.654–1.272)	0.587
Europe	1.100 (0.731–1.669)	0.637
Southeast Asia	0.602 (0.394–0.918)	0.019
Western Pacific	0.780 (0.577–1.054)	0.106

*Methods: The multivariate negative binomial regression model compared the timeliness of outbreaks first communicated by nongovernmental sources to those by governmental sources, while adjusting for geographic region and whether the outbreak occurred before or after public recognition of SARS. IRR, incidence rate ratio; SARS, severe acute respiratory syndrome; ref, reference value = 1. Reference categories: (1) source: governmental, (2) SARS: pre-SARS, (3) geographic region: Africa.

**Figure 2:**
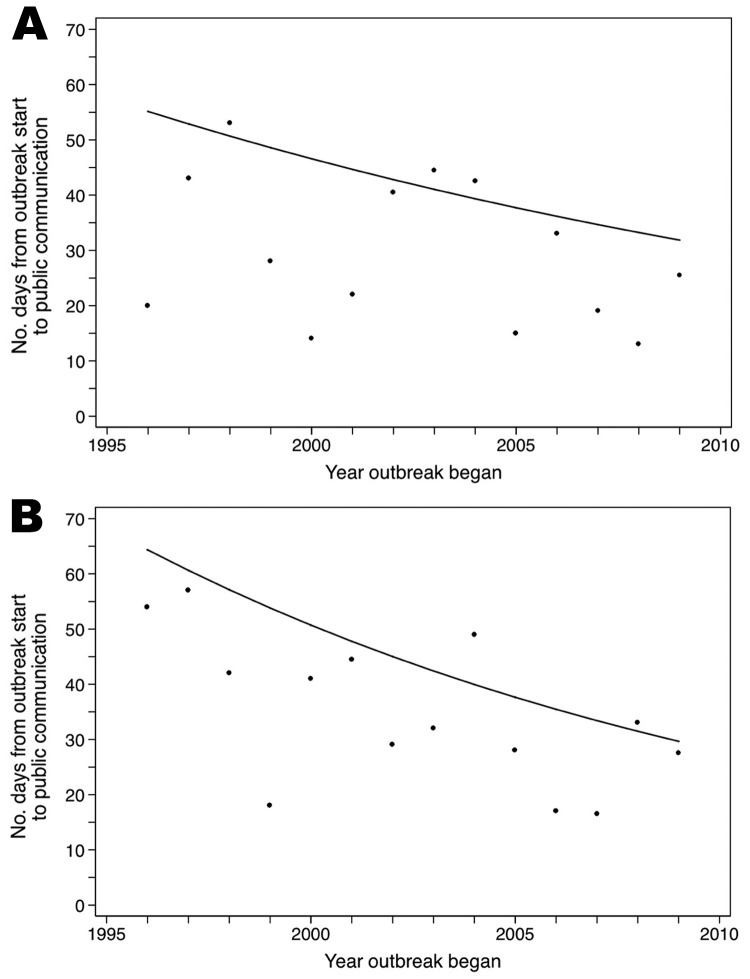
Median time (days) from the estimated start of an outbreak to its public communication for outbreaks reported by nongovernmental sources (A) and governmental sources (B), 1996–2009. Trendlines show average improvements over the study period.

Examination of temporal trends over the study period (Figure 2) showed that nongovernmental sources generally communicated outbreak signals to the public faster after 1996, although the trend did not reach statistical significance (IRR = 0.96, 95% CI 0.91–1.01). Governmental sources, in contrast, made significant improvements in lessening the time in which they publicly communicated initial outbreak signals (IRR = 0.94, 95% CI 0.91–0.97).

## Conclusions

Our data suggest that, from 1996 through 2006, outbreaks reported initially by nongovernmental sources were communicated publicly an average of 10 days earlier than those reported initially by governmental sources. Though the differences varied, nongovernmental sources tended to report outbreaks faster than governmental sources when we compared outbreaks before and after SARS, or by WHO-defined region. The lack of statistically significant differences in initial communication timeliness by source is probably attributable to a lack of statistical power rather than a lack of effect.

Our results also provide support for the International Health Regulations 2005 revisions that allow WHO to use unofficial information to request verification from member states. Slightly more than one-third of all unique infectious disease outbreaks in the WHO Disease Outbreak News during this 14-year period were initially reported by informal information sources.

Traditional governmental public health reporting mechanisms remain an integral source for outbreak information, accounting for almost two-thirds of all initial reports over this period. Our results also show that these sources made statistically significant improvements in reporting early warnings of outbreak threats more rapidly to the public, which might result in part from a shift toward automated, electronic methods that improve the timeliness of communication (*12**,**13*). It is possible that enhancements in nongovernmental outbreak reporting systems also contributed to improvements in governmental outbreak reporting timeliness over the study period, but we were unable to test this assumption with the current data.

This study has potential limitations. We encountered difficulty in selecting and consistently applying criteria to determine the initial source of public communication from ProMED-mail reports, which could have resulted in misclassification bias. Although other reporting systems that use informal information exist, they either lack a publicly available archive (for example, Global Public Health Intelligence Network) (*14*) or their database did not cover the entire study period (for example, HealthMap) (*15*). According to Heymann, et al., 65% of outbreaks recognized by WHO are first identified by informal sources (*4*), a proportion we did not find. Some outbreak reports were excluded because of missing data. We were able to internally validate the data that remained, but these exclusions limited the study’s statistical power. Finally, use of outbreak reports collected from the WHO Disease Outbreak News might limit the generalizability of our findings to all infectious disease outbreaks. Despite these limitations, our data highlight the value of nongovernmental sources as an integral resource for providing timely information about global infectious disease threats, and demonstrate the significant improvements in the timeliness of outbreak reporting made by governmental sources.
